# Muscle stiffness indicating mission crew health in space

**DOI:** 10.1038/s41598-024-54759-6

**Published:** 2024-02-20

**Authors:** Britt Schoenrock, Paul E. Muckelt, Maria Hastermann, Kirsten Albracht, Robert MacGregor, David Martin, Hans-Christian Gunga, Michele Salanova, Maria J. Stokes, Martin B. Warner, Dieter Blottner

**Affiliations:** 1 NeuroMuscular System & Signaling Group, Berlin Center of Space Medicine and Extreme Environments, 10115 Berlin, Germany, Institute of Integrative Neuroanatomy, Charité—Universitätsmedizin Berlin, Corporate Member of Freie Universität Berlin, Humboldt-Universität zu Berlin, and Berlin Institute of Health, 10115 Berlin, Germany, 10115 Berlin, Germany; 2 School of Health Sciences, University of Southampton, Southampton, UK; 3 Experimental and Clinical Research Center (ECRC) and NeuroCure Clinical Research Center (NCRC), Charité - Universitätsmedizin Berlin, Berlin, Germany; 4 Aachen University of Applied Sciences, Aachen, Germany; 5 Airbus US Space & Defense Inc. at JSC, Houston, TX USA; 6 JSC-SK KBR Wyle Services, Houston, TX USA; 7 Institute of Physiology, Berlin Center of Space Medicine and Extreme Environments, Charité—Universitätsmedizin Berlin, Corporate Member of Freie Universität Berlin, Humboldt-Universität zu Berlin, and Berlin Institute of Health, 10115 Berlin, Germany, Berlin, Germany

**Keywords:** Ageing, Physiology, Anatomy, Musculoskeletal system, Muscle, Tendons, Predictive markers, Prognostic markers, Predictive medicine

## Abstract

Muscle function is compromised by gravitational unloading in space affecting overall musculoskeletal health. Astronauts perform daily exercise programmes to mitigate these effects but knowing which muscles to target would optimise effectiveness. Accurate inflight assessment to inform exercise programmes is critical due to lack of technologies suitable for spaceflight. Changes in mechanical properties indicate muscle health status and can be measured rapidly and non-invasively using novel technology. A hand-held MyotonPRO device enabled monitoring of muscle health for the first time in spaceflight (> 180 days). Greater/maintained stiffness indicated countermeasures were effective. Tissue stiffness was preserved in the majority of muscles (neck, shoulder, back, thigh) but Tibialis Anterior (foot lever muscle) stiffness decreased inflight vs. preflight (p < 0.0001; mean difference 149 N/m) in all 12 crewmembers. The calf muscles showed opposing effects, Gastrocnemius increasing in stiffness Soleus decreasing. Selective stiffness decrements indicate lack of preservation despite daily inflight countermeasures. This calls for more targeted exercises for lower leg muscles with vital roles as ankle joint stabilizers and in gait. Muscle stiffness is a digital biomarker for risk monitoring during future planetary explorations (Moon, Mars), for healthcare management in challenging environments or clinical disorders in people on Earth, to enable effective tailored exercise programmes.

## Introduction

Human movements are performed habitually against gravitational forces (1G) on Earth^1^. During spaceflight, microgravity (µG) supports the astronaut’s body, removing the stimulus to maintain upright posture, with only low muscle activity required to perform daily functional tasks on the spacecraft. Astronauts are therefore subject to mission health risks related to disuse, such as muscle weakness and bone loss^2,3^, with up to a 20% decrease in skeletal muscle mass over one month^4^. To mitigate muscle loss and maintain muscle health, performance and crew safety^5^, astronauts on the International Space Station (ISS) perform a mandatory multi-modal programme using exercise hardware for approximately 2 h/day, 6–7 days/week^6,7^. Optimal effectiveness of exercise programmes requires iterative assessment to inform which muscles to target^8^. However, monitoring the effects of these countermeasures has only been documented pre-to-postflight, using technologies unsuitable for using inflight due to their size, complex to use, subject to interference, influenced by gravity or invasive, such as strength testing devices, electromyography, computed tomography, magnetic resonance imaging, muscle biopsies^7^. Whilst pre-/post- flight assessments provide indications of the distribution of muscle loss, they are not feasible for use onboard to inform tailored inflight exercise programmes. Inflight investigations of astronauts were called for by the European Space Agency Topical Team on Postmission Exercise (Reconditioning)^9^ to improve effectiveness of countermeasure exercise for successful prevention and reconditioning^10^. The work described here addressed the challenge of non-invasive inflight monitoring of muscle health using novel technology that is compact, accurate and involves suitable measurement protocols that meet crew requirements to their compliance (e.g. easy to use and rapid), thus advancing ability to make objective assessments of muscle properties inflight. Twelve astronauts were monitored throughout their mission cycles to establish a robust protocol for assessment in the space environment to gain a better understanding of the mitigating effects of current exercise programmes on the muscular system in microgravity. Specifically, it was unknown whether effects of exercise were of a uniform distribution or selective in nature, in terms of extent and time course, possibly requiring more targeted exercises in terms of more personalized protocols than currently performed. Further sites were examined than in previously reported pre-to-postflight studies.

This work has direct relevance for people living with conditions affecting muscles on Earth. Physical inactivity in everyday modern life has become a pandemic and major contributor to serious long-term conditions, such as cardiovascular, metabolic and musculoskeletal disorders^11^. Muscle loss after injury, and joint and muscle disorders pose further health risk for people of all ages but particularly for older people in danger of losing their physical independence^12^. Exercise is a well-accepted preventive measure to mitigate these health risks^13^ but, as mentioned above, exercises need to be targeted at the most relevant muscles to be optimally effective^8^. Whilst a greater range of technologies is available on Earth than in space, certain environments pose a challenge to using accurate assessment tools, such as measuring muscle strength, due to factors such as cost of equipment, time, need for expertise, practical feasibility, space etc. Such environments lacking suitable means for monitoring muscle health outside the laboratory include: physical rehabilitation facilities, clinics in hospitals and general practice; remote facilities without clinical expertise, such as in rural communities and in people’s homes. Astronauts are considered to be in excellent health as part of their job description in space and perform a mandatory complex multi-modal programme using exercise hardware for up to 2–2.5 h/day for 6-7 days/week^6,7^ to maintain muscle health, inflight performance and crew safety^5^. However, recent astronaut twin studies showed health issues and ageing processes occurred much faster in space than on Earth, suggesting the space environment is a unique laboratory to investigate change in health status and deconditioning^14^.

Human musculoskeletal health is a complex interplay between several intrinsic factors (e.g. fiber type characteristics^15^, capillary density and blood perfusion^16^, metabolism^17^, sensorimotor adaptation^18^, fluid shift and tissue hydration^19^, intramuscular connective tissue^20^, and external factors, such as high protein intake in nutrition^21^. Together, these factors influence intrinsic biomechanical muscle properties^22^, such as stiffness, detectable by natural damped oscillations of soft biological tissue layers^23,24^. Intrinsic mechanical viscoelastic properties of the myofascial system support muscle force transmission and uphold muscle tension at rest independently of contractile activity^25,26^. Examples of factors that would increase stiffness include: type II (anaerobic) muscle fibers^15^, reduced blood perfusion, reduced hydration^19^, high proportion of intramuscular connective tissue^20^. The converse would reduce muscle stiffness, e.g. type I (aerobic fibers) etc.

Passive muscle stiffness is known to change in the short-term in every-day life, increasing as force of muscle contraction increases, after physical activity, with increases in passive muscle length as joint angles increase (and vice versa)^27^, and stiffness reduces as temperature increases^28^. Assessing passive stiffness therefore requires standardized recording conditions to ensure robust protocols^27^. Passive stiffness also increases over time with ageing^29^, resistance training^30^, muscle diseases, e.g. Duchenne muscular dystrophy^31^, and in neurological disorders, e.g. Parkinson’s disease^32^ and stroke^33^. Monitoring changes in muscle stiffness can thus be considered a suitable indicator of muscle health status in patients, as well as for monitoring effects of microgravity and training in astronauts during prolonged spaceflight, provided standardized protocols are used^27^.

Recent spaceflight analogue studies (dry immersion, bed rest) monitored passive muscle stiffness indices in healthy volunteers using a non-invasive MyotonPRO device with or without exercise as a countermeasure^34,35^. The smartphone-sized gauge is a hand-held, non-invasive digital palpation device measuring biomechanical tissue properties, such as stiffness (N/m) of superficial structures (muscle, tendon and fascia). The measured indices are based on computing reflected damped natural oscillations by the device`s algorithm^36^. Myoton technology has been shown to be a valid (against shear wave elastography)^37^, reliable and feasible method to collect data in clinical studies^38,39^, in healthy males and females^29,40^, and robust during brief periods of weightlessness conditions during parabolic flight^35^. Known characteristics of muscle stiffness that indicate muscle strength have been demonstrated using Myoton technology, such as increased stiffness with force of contraction^41^ and greater stiffness in males than females^29^.

The present MYOTONES experiment (2018–2023) aimed to monitor muscle health quantitatively for the first time in spaceflight via passive stiffness (N/m) recordings as digital biomarker from surface soft tissues (muscle, tendon and fascia). Specific measurement points on the astronauts´ bodies were monitored using a space-qualified Myoton device throughout a mission cycle including preflight, 4–11 months stay on the ISS and up to 3 months postflight. The purpose was to increase understanding of the effects of microgravity on human musculature and preservation effects of exercise countermeasures, to inform more targeted interventions. It is hypothesized that maintenance of muscle stiffness throughout a space mission would indicate that countermeasures mitigated loss of muscle function due to prolonged microgravity. Routine monitoring and targeted interventions will become vital for missions beyond low earth orbit (LEO), ensuring safe performance of tasks during long-duration exploration, such as on the Moon and Mars. Insights from space could translate to better understanding and management (exercise protocol design) to alleviate poor muscle function and performance in people on Earth in relation to ageing and healthcare in remote areas, sojourns in extreme environments, and various clinical conditions affecting muscle health that present as major challenges to mobility and independence.

## Results

### Myoton data: tissue stiffness (N/m)

**Figure 1:**
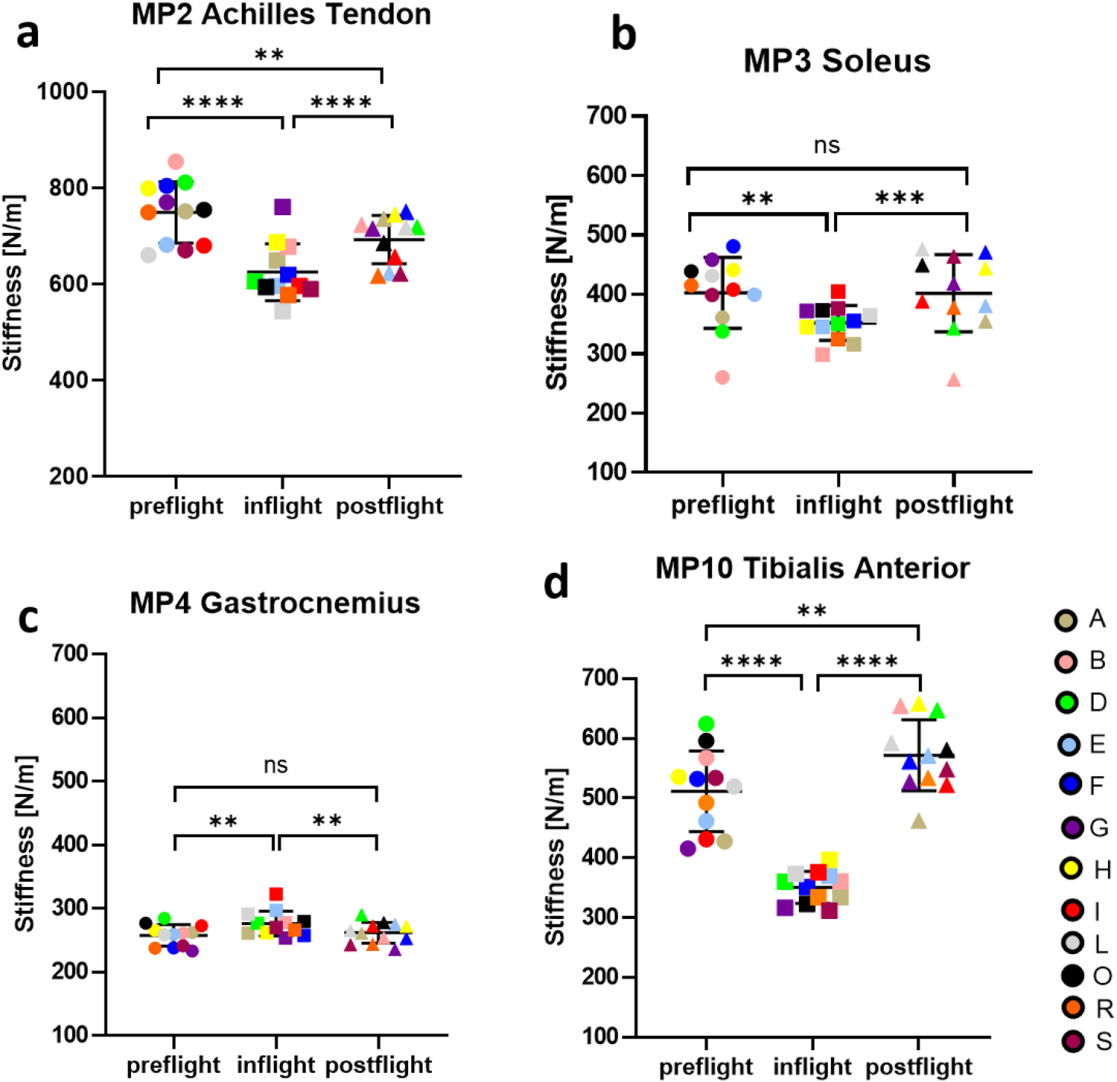
Lower leg muscular stiffness. Mean Stiffness [N/m] of: (**a**) MP2 Achilles Tendon; (**b**) MP3 Soleus; (**c**) MP4 Gastrocnemius medialis; (**d**) MP10 Tibialis anterior. Mean values for individual astronauts (A-S) colour coded for visualization of intersubject variability; mean values (for each test condition) of n = 12 with standard deviation for preflight (BDC L-180 and BDC L-60, circle data points), inflight (FD5-15, FD30-60, FD121-150 and, R-10, square data points) and postflight (R + 1, R + 5, R + 30, and R + 105, triangle data points); *p < 0.05, **p < 0.01, ***p < 0.001, ****p < 0.0001; *ns* non significant.

**Figure 2:**
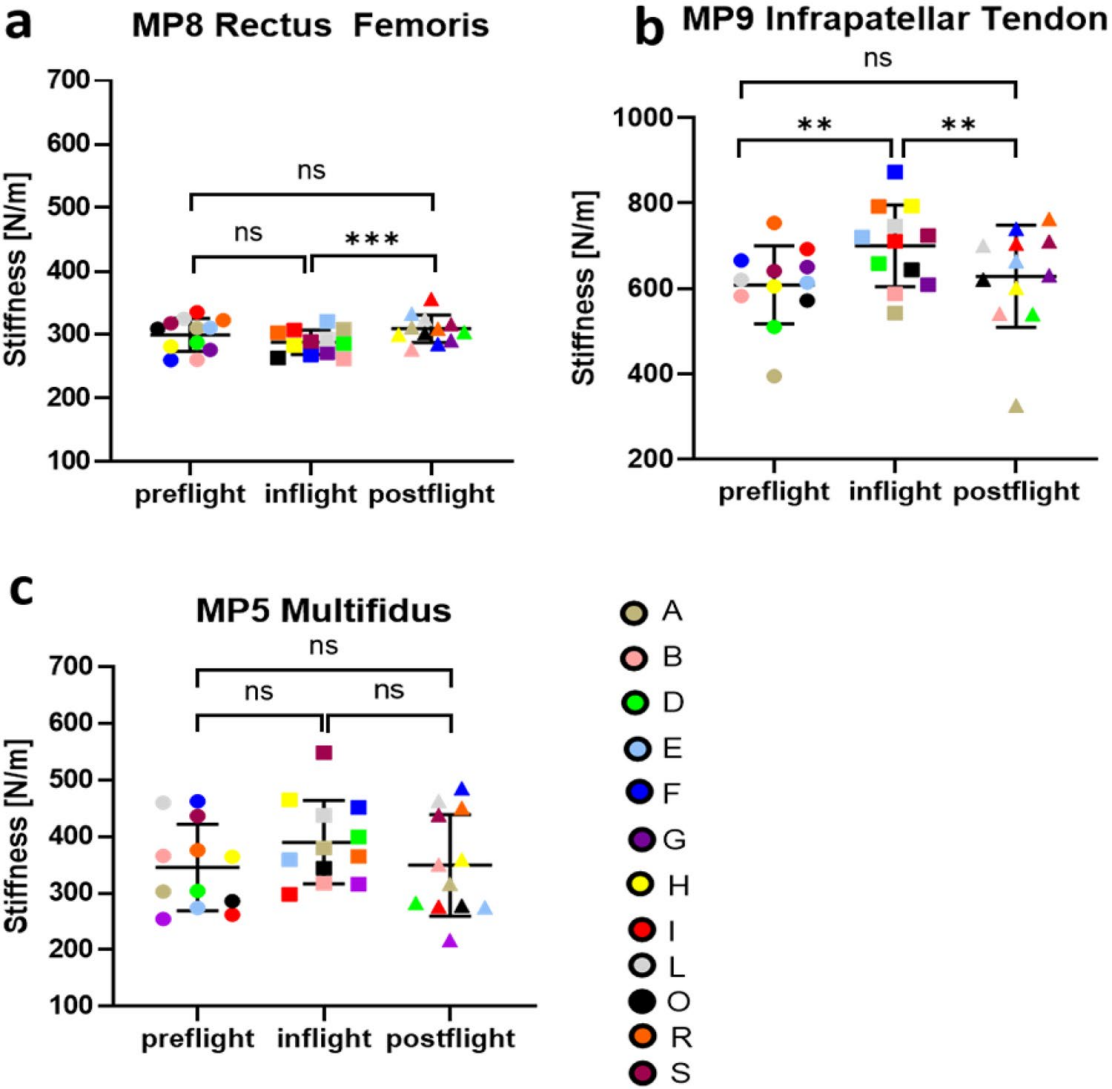
Thigh and lumbar back muscular stiffness. Mean Stiffness [N/m] of: (**a**) MP8 Rectus Femoris; (**b**) MP9 Infrapatellar tendon; (**c**) MP5 lumbar Multifidus. Mean values (for each test condition) for individual astronauts (A-S) colour coded for visualization of intersubject variability; mean of n = 12 with standard deviation for preflight (BDC L-180 and BDC L-60, circle data points), inflight (FD5-15, FD30-60, FD121-150 and, R-10, square data points) and postflight (R + 1, R + 5, R + 30, and R + 105, triangle data points); *p < 0.05, **p < 0.01, ***p < 0.001, ****p < 0.0001; ns = non significant.

Tissue stiffness [N/m] recordings of muscle, tendon or fascia, obtained using a MyotonPRO device (Myoton AS, Estonia) from 10 measurement points (MPs) over the skin are presented below according to anatomical and functional groups (Supplementary Fig. S1). Stiffness measurements varied across the time periods from preflight, inflight and postflight, Significant changes are presented in Figs. 1 and 2. For other skin measurement points (MP1, MP6 and MP7) refer to Supplementary Material (Supplementary Fig. S2).

### Lower leg (MP2, MP3, MP4, MP10)

There were statistically significant differences for all lower leg measurement points between time periods, as determined by one-way ANOVA with repeated measures (MP2 F[2,117] = 27.21, p < 0.0001; MP3 F[2,117] = 10.09, p = 0.0001; MP4 F[2,117] = 9.038, p = 0.0002; MP10 F[2,117] = 107.9, p < 0.0001). Calf muscles (Soleus [MP3] and medial Gastrocnemius [MP4]) showed inverse changes in stiffness relative to one another during microgravity, with Soleus decreasing in stiffness inflight (p = 0.0029; mean difference 50.58 N/m < minimal detectable change [MDC] 141 N/m of repeated measurements)^40^ and Gastrocnemius increasing in stiffness (p = 0.0011; mean difference 18.62 N/m < MDC 28 N/m). In both muscles, stiffness recovered postflight to preflight values (MP3 pre vs post ns, p = 0.9990; MP4 pre vs post ns, p = 0.6842) (Fig. 1b/c). The common tendon for the two calf muscles (Achilles Tendon [MP2]) decreased in stiffness pre- to inflight (p < 0.0001; mean difference 124.1 N/m > MDC 59 N/m) but recovery values did not reach preflight values (pre vs post p < 0.0001; mean difference 56.31 N/m < MDC 59 N/m) (Fig. 1a). The calf muscles’ antagonist Tibialis Anterior (MP10) decreased significantly in stiffness inflight in all astronauts compared to pre- (p < 0.0001; mean difference 149 N/m > MDC 89 N/m) and postflight (p < 0.0001; mean difference 209.3 N/m > MDC 89 N/m). Postflight values were significantly higher than preflight values (p = 0.0027; mean difference 60.23 N/m < MDC 89 N/m) (Fig. 1d).

### Thigh (MP8, MP9)

The knee extensor Rectus Femoris (MP8) muscle stiffness did not change significantly pre- to inflight, with only a slight decrease (ns, p = 0.1705; mean difference 11.66 N/m < MDC 34 N/m^29^) with an increase upon return (in- to postflight p = 0.0003; mean difference 21.20 N/m < MDC 34 N/m^29^) (Fig. 2a). The corresponding tendon (Infrapatellar Tendon [MP9]) showed inverse change with statistically significant increases in stiffness inflight (pre- to inflight p = 0.0030; mean difference 95.69 N/m > MDC 51 N/m; inflight to postflight p = 0.0044; mean difference 75.47 N/m > 51 N/m). Recovery values reached preflight values (ns, p = 0.7583) (Fig. 2b).

### Lower back (MP5)

There were no significant differences found on ANOVA for the Multifidus muscle at MP5 (F[2,116] = 2.87, p = 0.0609) (Fig. 2c). The muscle showed no changes from preflight to during the inflight period or from inflight to postflight (Fig. 2c). There were, however, some differences between time periods observed when mean differences were compared to MDC values (MDC 22.8 N/m^42^; pre vs inflight 41.12 N/m > MDC; in vs postflight 37.24 N/m > MDC; pre to postflight 3.88 N/m < MDC;).

### Neck and shoulder muscles (MP6, MP7)

There were no significant differences for neck (MP6) or shoulder (MP7) muscles throughout the mission cycle. The ANOVA results for the paraspinal Splenius Capitis neck muscle (MP6) were: F[2,117] = 2.024, p = 0,1367; MP7 F[2,117] = 0.2965, p = 0.7440, with a slight pre- to inflight decrease in stiffness below the MDC (ns p = 0.7145; mean difference 8.9 N/m < MDC 59 N/m). The shoulder muscle Deltoideus Anterior (MP7) showed only a trend in change inflight (ns p = 0.9483; mean difference 3.36 N/m < MDC 41 N/m), with a wide variability between crew members. Recovery values reached preflight values for both muscles (Supplementary Fig. S1).

### Plantar fascia (MP1)

The fascia on the sole of the foot (MP1) showed no statistically significant change in stiffness from preflight to inflight or in postflight recovery (F[2,117] = 2.927, p = 0.0575), with wide participant variability. Mean differences across time periods were all below MDC values (MDC 47 N/m; pre vs in 34.37 N/m; in vs post 0.91 N/m; pre vs post 33.46 N/m) (Supplementary Fig. S1).

### Linear mixed model analysis inflight and posflight

**Figure 3:**
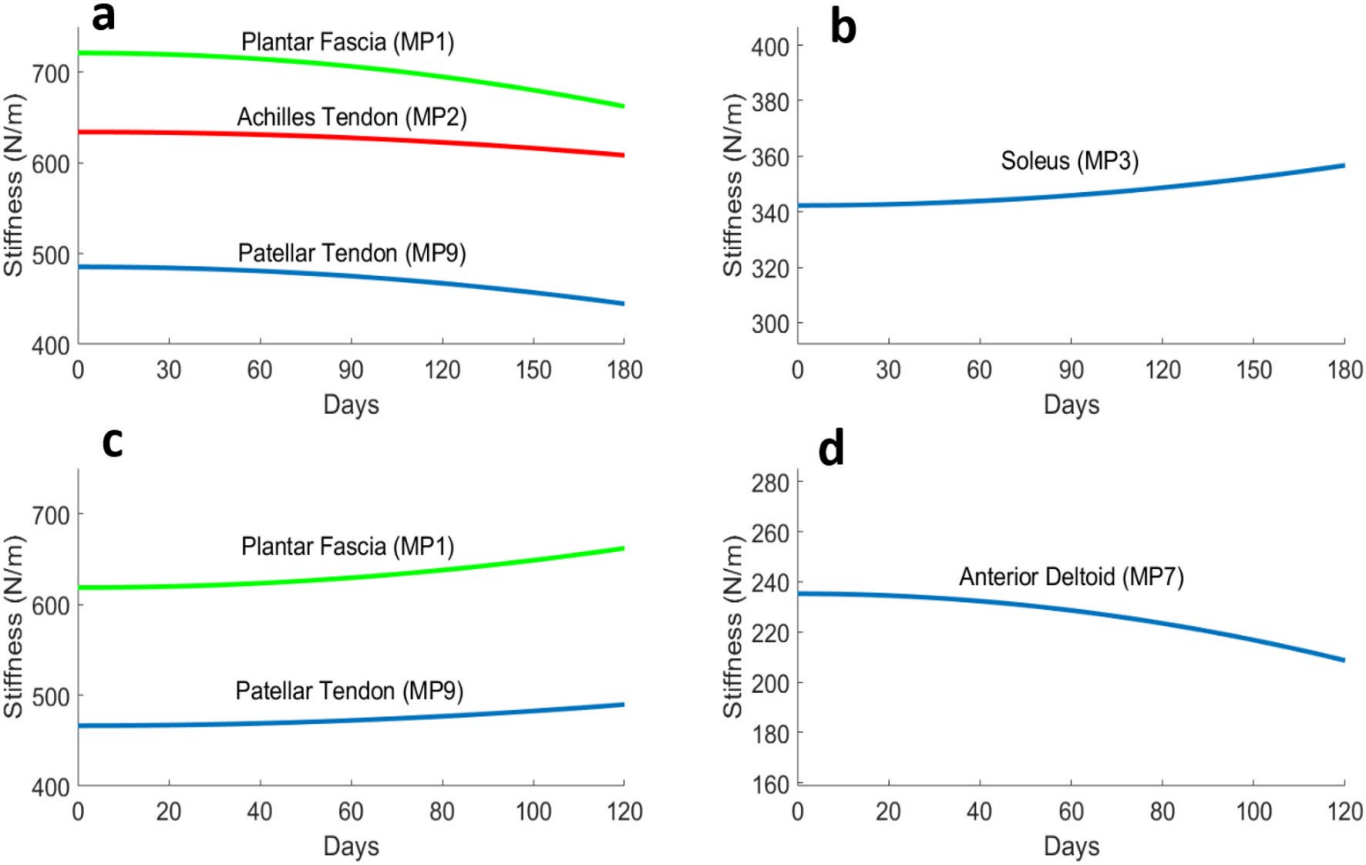
Regression model for muscular tissue stiffness changes inflight vs postflight (each n = 12). Upper panels (inflight) (**a**) Plantar fascia (MP1), Achilles tendon (MP2) and Patellar tendon (MP9) (**b**) Soleus (MP3). Lower panels (postflight) (**c**) Plantar fascia (MP1) and Patellar tendon (MP9); (**d**) Anterior Deltoid (MP7).

Visualisation of the case-wise relationship between number of days and stiffness indicated a quadratic relationship for both inflight and postflight changes for some sites of the muscular system (Fig. 3).

### Inflight changes in stiffness

A significant quadratic fixed-effect relationship was found between number of inflight days and stiffness for MP1 (p = 0.002), MP2 (p = 0.036), MP3 (p = 0.02) and MP9 (p = 0.04). The measurements sites related to tendons/ligaments (i.e. MP1, MP2, and MP9) all demonstrated a decrease in stiffness over time (Fig. 3a). The soleus muscle site (MP3) demonstrated an increase in stiffness over time (Fig. 3b). The remaining six sites (Supplementary Fig. S5) did not demonstrate a significant quadratic relationship during inflight conditions. Please see Supplementary Table S1 for data supporting the p-values.

### Postflight changes in stiffness

A significant quadratic fixed effect relationship was found postflight for MP1 (p = 0.05), MP7 (p = 0.02) and MP9 (p = 0.03). The plantar fascia (MP1) and patellar tendon (MP9) sites demonstrated an increase in stiffness over time (Fig. 3c). The measurement related to the anterior deltoid (MP7) demonstrated a decrease in stiffness over time (Fig. 3d) following recovery postflight.

### Skin temperature from thermal imaging (FLIR camera system)

There were no significant changes in skin temperature over time at any of the MPs, indicating stable experimental conditions. Skin temperature measured during baseline data collection (BDC) sessions varied between MPs, with MP1 having the lowest skin temperature (29.0 °C) and MP6 the highest (35.4 °C) (Supplementary Table S2). All measurements of skin temperature lay within the physiological range^43^.

### Subcutaneous tissue thickness from ultrasound imaging

There were no significant changes in subcutaneous tissue thickness documented over time for each of the 10 MPs. (Supplementary Figs. S2 and S3).

## Discussion

The MYOTONES experiment was the first to collect data on muscle health (passive stiffness during full body unloading in microgravity) in astronauts during an entire space mission cycle involving inflight measurements at four time points on the ISS. The study protocol was designed for autonomous and compliant use by astronauts onboard the ISS as a pioneering inflight health monitoring tool for real-time muscle status and postflight rehabilitation assessment. The technology used in the present study has improved ability to examine distribution of muscle loss. By using passive muscle stiffness [N/m] recordings as digital biomarkers, we showed that most of the skeletal muscles and tendons studied are targeted effectively by inflight countermeasure exercise to mitigate microgravity-induced disuse, with the exception of Tibialis Anterior and the deep calf muscle Soleus. This evidence of the feasibility of selectively obtaining human muscle health data relatively simply within the unique space environment could pave the way for translation to enhanced global healthcare, through monitoring in everyday life, sports, aging populations and people living in challenging environments, as well as various clinical settings for musculoskeletal and neurological disorders on Earth.

The present novel insights indicate that a countermeasure exercise programme performed onboard the ISS generally preserves passive stiffness in muscle structures at most sites measured (shoulder, neck, back, thigh), as changes inflight showed only marginal differences from pre- and postflight measurements. Importantly, the Tibialis Anterior muscle (prime dorsiflexor, ankle joint stabilizer^44^, vital in human gait on Earth, showed reduced stiffness inflight in all 12 mission crew members studied in spite of routine inflight countermeasures currently available on the ISS^45^ (T2, treadmill-2; ARED, advanced resistive exercise device; CEVIS, bicycle ergometer). This selective lack of preservation of stiffness, suggesting muscle loss/weakness, in an important muscle for gait is a new and unexpected finding, which requires consideration for ISS astronauts and longer-duration missions to Deep Space.

As part of the prime plantarflexor calf triceps surae (Soleus and Gastrocnemius) both antagonists to Tibialis Anterior, only Gastrocnemius (medial head) showed increased stiffness (Fig. 1c) inflight, in contrast to Soleus which showed a decrease (Fig. 1b). These inverse changes in stiffness may be due to the known muscle tissue diversity, i.e. fibre-type composition (Soleus predominantly slow fibers; Gastrocnemius mixed-fast-contracting fibres)^46^, and their functional characteristics^47^. Decreased stiffness in Soleus may be due to an altered force–length relationship (operating length) normally observed in human gait^27,48^ which is likely compromised by gravitational unloading causing the plantarflexed ankle joint position of the relaxed astronaut´s body at rest (semi-squatting) in microgravity. This second novel observation from the space environment of inverse changes within the calf musculature has not been reported in previous space analogues, such as bed rest studies^49,50^ but reiterates the need for standardized measurement conditions and appropriate body positioning during measurement, as these are key factors for reliable sampling and meaningful data interpretation^27^. Although Soleus muscle stiffness decreased compared to preflight, it did increase gradually over time, as demonstrated by a significant change in stiffness in a quadratic relationship with number of days spent on board the ISS. Therefore, it is possible that the countermeasures to mitigate the loss of muscle mass in microgravity help to preserve stiffness of the Soleus muscle to some extent. It is furthermore hypothesized that in space, Gastrocnemius at least partially overtakes function of the entire calf muscles (indicated by increased stiffness) with or without inflight countermeasures. Their mutual Achilles Tendon showed decreased stiffness inflight compared to preflight, which was also demonstrated in a quadratic relationship with number of days spent on board the ISS. The cause of decrease in stiffness is again likely due to the ankle joint of the fully unloaded body in microgravity being at a higher (plantaflexed) resting angle than on Earth. This results in less stretching and thus reduced storing and releasing elastic strain energy of the calf muscle–tendon unit in space than usually seen in terrestrial walking, running and jumping^51,52^ on Earth as previously shown^53^. Monitoring Achilles tendon stiffness is critical for astronauts, as well as for healthy people of all ages on Earth^54^, since sudden reloading, such as gravitational force transitions and strenuous activity, can result in Achilles Tendon injury or even rupture^55^. Of particular note is that stiffness of the Achilles Tendon postflight was significantly lower than preflight and there was no significant relationship with stiffness and number of days post flight, in contrast to other tendons. Therefore, the risk of rupture for Astronauts might be high on strenuous activity, without appropriate reconditioning. The Achilles tendon rehabilitation process is relatively long and is known to have a high risk of tendon rerupturing^56^. Myoton technology used in the present study may prove to be an effective tool to monitor Astronauts’ reconditioning after return from spaceflight but also the tendon’s stiffness to evaluate healing processes and to adapt required therapeutic decisions on the magnitude of reloading of the foot in relevant clinical settings^57^. Reloading on return to gravity will also be vital to consider for partial gravity conditions during extravehicular activities in future planetary explorations (Moon/Mars)^4^. The exercise countermeasures inflight aim to minmise this risk of injury during realoading.

In contrast to the Achilles tendon, the Infrapatellar Tendon (tendinous link of Quadriceps Femoris muscle to the tibial bony tuberosity via the knee cap) showed increased stiffness in microgravity as the habitual lower limb semi-squatting posture in space is knee flexion, resulting in a stretched patellar tendon. Stiffness of Rectus Femoris (superficial mid-head of Quadriceps Femoris) remained unchanged in crew members onboard the ISS. The Rectus Femoris is a two-joint muscle (hip and knee)^58^ and hip flexion, which is the main position adopted for the crew members’ free-floating bodies in microgravity, probably counters the stretch effect of knee flexion. The high intensity inflight exercise usually performed by crew members on the advanced resistive exercise device (ARED) specifically targets the prime knee extensor, resulting in effectively maintaining Quadriceps muscle strength and stiffness^59^. However, it is important to note that there was a negative relationship in stiffness and number of days spent on the ISS for the Infrapatellar tendon inflight, so although stiffness increased pre- to inflight, it gradually declined inflight. As with the Achilles tendon, careful monitoring is important to track changes and identify potential risks for rupture or other injuries in the Space Medicine context.

Low back pain is a well-known problem following extended immobilization^60^ and in astronauts in spaceflight^61^. Lumbar Multifidus atrophy and flattening of the lumbar lordosis have been reported in astronauts after long-duration spaceflight^62^. The higher stiffness of Multifidus observed inflight in the present study reduced significantly upon return, contrary to findings in a dry immersion study which reported reduced stiffness of the lower lumbar Erector spinae^63^, although a higher stiffness may be explained by inflight stretch of the muscle due to the flattening/elongation of the lower spine and a compression of the spine upon reloading^64^. Low back pain is a major health issue on Earth due to high workload and seen in occupational medicine cohorts, such as nurses, delivery workers, office workers due to prolonged sitting, with less activity to maintain strength of the dorsal paravertebral muscle system and reduce strain on the spine itself^65^. Specifically, the Erector Spinae muscles extend and rotate the spine and Lumbar Multifidus stabilizes the spine (the latter monitored in the present study). Muscle stiffness detected by the Myoton technique has the potential to be a clinical indicator of spinal muscle health in order to quantitatively assess back pain syndromes in routine medical examination in real-time.

The variability in muscle stiffness between participants for gastrocnemius and rectus femoris was smaller than compared with other muscles and tendons (Figs. 1 and 2). This observation existed across the mission cycle, so was not related to effects of microgravity or countermeasures. Pre-flight data for Rectus Femoris (Fig. 2a), for example, were similar to mean and standard deviation values for 61 healthy young adults (males 292 ± 36; females 233 ± 35) reported by Agyapong-Badu et al.^29^. A possible technical reason for differences in variability may relate muscle size and the relative effects of force of the mechanical impulse from the Myoton device, which is standardized across muscles. The Rectus Femoris and Gastrocnemius are larger than most of the other muscles studied and inter-subject variability in body size would affect smaller muscles more than larger muscles. However, the Gastrocnemius and Soleus muscles are similar in size, yet their inter-subject variability differed (Fig. 1). Perhaps the homogeneity of muscles, e.g. in terms of fiber-type composition, is reflected in variability in their physiology and behavior.

Future long-duration missions into Deep Space will require compact design spacecraft with limited cabin space, providing less opportunity to perform countermeasure exercise using similar devices to those currently available on the ISS. Impaired muscle function increases the risk of poor performance during planetary excursions in partial gravity (e.g. Moon, Mars) but also increases the risk of falling upon return to Earth’s gravity (1G). The variability of changes between astronauts observed in the present study suggests that personalized countermeasure exercises targeting specific muscle groups will be required during future long-duration missions to enable safer human planetary exploration^66^. Monitoring muscle health will be vital in this as yet unknown Deep Space environment and future countermeasure protocols need to be adjusted with respect to newly identified mission health risks of astronauts as shown in this work. In particular, close monitoring will be crucial for future Deep Space and Planetary Exploration where gait and postural stability are utmost requirements for human motion in altered gravity conditions (Moon, Mars), as well as for faster recovery on return to Earth.

Lessons from the present data regarding rapid, simple, non-invasive monitoring of muscle health have implications for assessing human health on Earth outside a laboratory environment, as this is not accessible for routine patient care. Such settings include: open field research; patients in remote situations, such as General Practitioner consultations, physiotherapy treatment sessions, hospital outpatient clinics and wards, and patients’ homes. Critical care is a particularly relevant setting for continued monitoring, where patients’ muscles can change rapidly on a daily basis^67^. Such monitoring (without the need for biopsy) would help more targeted treatments, as proposed in this work for astronauts on long-duration missions. Treatments and assessments of older people may also benefit from monitoring muscle health since sarcopenia (age-related muscle wasting) shows similar traits to muscle loss in microgravity^68^ and stiffness changes with ageing have already been documented with Myoton technology^29^.

Lessons learned from spaceflight highlight some critical requirements for meaningful data acquisition from resting human muscle under normal 1G gravitational conditions on Earth. Deviation from the following recording conditions are likely cofounders of robust data acquisition using Myoton technology, some of which have been documented^27^: (i) fully relaxed body position (standing, sitting, lying); (ii) limb dominance (left/right); (iii) accurate location of skin measurement points (in the middle of a relaxed muscle belly/tendon length on superficial anatomical muscle/tendon units of interest); (iv) standardized joint positioning/angle (identical passive muscle lengthening) particularly when single-joint muscles (e.g. Soleus) versus two-/multiple-joint muscles (e.g., Biceps Brachii, Rectus Femoris, Gastrocnemius) as muscle of interest; (v) variable body composition/adiposity (excess of subcutaneous tissue/fat deposits over muscle of interest in obesity); (vi) avoidance of prior physical activity, which can increase muscle stiffness; (vii) and consistent time of day (diurnal variability) of measurements, as various aspects of muscle function vary, e.g. changes in resting muscle characteristics across the day were reported in professional athletes^69^.

Study limitations imposed by space-related research include: (i) lack of ability to perform electromyography (EMG signal silence to check for absence of electrophysiological input/muscle activity) during sessions inflight (operational constraint)^70^; (ii) the relatively low number of astronauts (8 male vs. 4 female) prevented sound analysis of sex differences, as well as differences between novice astronauts and those who had flown previously (statistical constraint); (iii) slightly variable flight schedules and mission durations between astronauts prevented presentation of data at particular time points and, instead, mean values for time periods were used to enable comparisons across mission duration (statistical constraint). To account the limitation in this analysis, the separate linear mixed models analyses in Fig. 3 demonstrate changes over time both inflight and postflight; (iv) muscles examined was limited to 10 MPs due to time constraints but in principle, there is no limitation for the Myoton protocol to include many other superficial muscle groups of interest and relevance to more targeted exercise. Selection of the 10 MPs was based on relevance to movement/postural control on Earth, on a previous bed rest protocol^50^ and knowledge of the behavior of various muscles in health and dysfunction.

The assessment protocol and definition of passive muscle stiffness as a muscle health indicator from the space environment could be helpful for many health professionals undertaking clinical assessments, and widespread uptake may result in a step-change for enhancing healthcare in neuro-musculoskeletal and geriatric medicine, rehabilitation and precision medicine on Earth. For example, assessment of stiffness and other muscle characteristics is very helpful in the management of neurological disorders such as Parkinson Disease (PD)^71^ and stroke^38^. Muscle stiffness measured conventionally under laboratory conditions requires cumbersome, expensive equipment but laboratory environments are not accessible for routine clinical care. Clinical assessment of muscular stiffness involves subjective protocols comprising manual palpation and evaluation of passive movements, and rating stiffness as mild, moderate or severe. As demonstrated in the astronaut study described, Myoton offers objective non-invasive measurement suitable for clinical environments to develop clinical scales, and for more accurate and sensitive evaluation of effects of treatments such as drugs^71^ and brain stimulation as in PD^32^. Potential future applications also include home monitoring of drug effects, analogous to self-testing blood in diabetes.

The MYOTONES study brings a new dimension to translating the technology to clinical assessment through training novice users by remote guidance via teleconference. An example of such impact was realised with physiotherapists in Ghana who were guided remotely to establish reliability of using Myoton in PD patients^72^.

Areas where muscle strength cannot be measured due to: lack of equipment; pain from traumata/injuries, joint disorders (rheumatoid and osteoarthritis); during surgical interventions; or lack of cognition, such as in intensive care patients and in frail older people at risk of losing physical independence^29^; are very promising examples of novel applications of the space-tested Myoton technology and protocol. Myoton also enables selective changes within a muscle group to be detected (e.g. the calf), which strength testing does not permit.

We conclude that stiffness was preserved in the majority of muscle structures studied but selective loss of stiffness of key muscles required for gait, despite daily inflight countermeasures, indicates more targeted exercises are required for effective countermeasures in space. Our work has contributed to the body of evidence for potential to translate Myoton technology to multiple other settings on Earth, from physical conditioning monitoring in remote areas or in extreme environments^73^ to clinical rehabilitation enabling more targeted and efficiently tailored interventions. These applications based on muscle stiffness as digital biomarker open up new avenues for improved health status management in future Human Deep Space Exploration and for people on Earth.

## Methods

### General study information

The MYOTONES project (2018–2023) involved 12 long-duration mission astronauts (male n = 8, female n = 4) from National Aeronautic and Space Administration (NASA), European Space Agency (ESA) and Japan Aerospace Exploration Agency (JAXA) throughout their mission cycle, which included 4–11 months inflight onboard the ISS. A mission cycle encompassed two preflight (on the ground preconditioning phase; BDC L-180 and BDC L-60), four inflight (2 sessions in 1st and 2 sessions in 2nd half of the mission onboard ISS; FD5-15, FD30-60, FD121-150 and, R-10), and four postflight sessions (recovery/reconditioning; R + 1, R + 5, R + 30*,* and R + 105), with the earliest session at 254 days before launch (baseline data collection, BDC) and the latest 123 days after return (for experimental timeline see Supplementary Fig. S4).

Procedures involved non-invasive data collection with digitized Myoton technology on the 10 pre-selected individual skin measurement points (superficial muscle/fascia/tendon), ultrasound imaging by remotely controlled B-mode ECHO system provided by ESA (to measure subcutaneous tissue thickness at skin MPs to aid interpretation of Myoton recordings), and skin surface temperature detection via FLIR ThermoCam system (see below) at skin MPs (pre/postflight temperature range check; see Supplementary Table S1).

### Anatomical structure data collection

Myoton measurements and ultrasound imaging of 10 skin measurement points (MP) were performed on: Plantar Fascia [MP1], Achilles Tendon [MP2], Soleus muscle [MP3], Gastrocnemius Medialis muscle [MP4], Lumbar Multifidus muscle [MP5], Splenius Capitis muscle [MP6], Deltoideus Anterior muscle [MP7], Rectus Femoris muscle [MP8], Infrapatellar Tendon [MP9], Tibialis Anterior muscle [MP10]. Due to contraints in crew time and to aid compliance, the inflight protocol was restricted to these 10 MPs reflecting both extensors and flexors, as illustrated in the MYOTONES body chart (Supplementary Fig. S5). Precise anatomical locations are described in detail elsewhere^40^.

### Myoton technology and protocol

The principle of Myoton technology (Myoton AS, Estonia)^36^ is based on natural damped oscillations of biological soft tissue. The Myoton device produces recordings of five parameters (stiffness, non-neural tone, elasticity, relaxation, creep) but stiffness was selected for a mechanical dynamic response and as digital biomarker for the present study, as it is a well-understood tissue property and Myoton recordings have been validated using a multi-layered phantom tissue model^23^ and in human muscle^37^. Inter and intra-rater reliability have also been established in healthy participants for all body measurement point (MP) sites relevant to the MYOTONES protocol^29,40,42^. We did not use electromyography (EMG) during the Myoton sessions to examine values at rest (silent EMG signal)^70^ due to several operational constraints (acknowledged in study limitations in the discussion).

After positioning the Myoton device perpendicular to the skin, the rounded tip of the probe applies a brief (15 ms), low force (0.4 Newton [N]) mechanical impulse with a constant preload (0.18 N). The present study analyzed passive muscle stiffness (S) recorded by damped oscillations as numerical parameters [N/m] of superficial skeletal muscle, tendon and a fascia, which is a meaningful biomechanical characteristic currently best understood in relation to soft biological tissue structure and composition^74^. Stiffness (S) is a measure of a tissue’s ability to resist an external force that modifies its shape and is calculated using the formula: S = a_max_ · m_probe_/ΔI; a = acceleration, m = mass of measurement mechanism, ΔI = maximum displacement, reflected as newton-meter [N/m] calculated by internal device algorithm (MyotonPRO User Manual rev17, 14th Nov. 2017). For each MP, five impulses were applied and a mean value was calculated. Coefficient of variation (CV) for the five impulses was accepted if it was lower than 3% (%), which usually only required one measurement set of impulses but measurement was repeated if the CV was > 3%. Data sampling on the ground was performed in a dedicated quiet room with the participant completely relaxed, according to a standardized protocol (sitting or lying on guerney with knee and ankle roll support at 10 degrees knee/ankle flexion), or in spaceflight with full body relaxation (unloaded semi-squatting position) onboard ESA´s Columbus module of the ISS.

For baseline data collection (BDC) sessions the MyotonPRO was used. For inflight sessions, a space-qualified MyotonPRO device (OHB Space Systems Inc., Bremen, Germany) was used (with manual ON/OFF switch); launched by NASA contractor SpaceX (Falcon 9, CRS rocket) to the ISS.

### Skin thermal imaging

Body and skin temperature are critical to a muscle’s biomechanical properties^32^. To monitor skin temperature at MPs, a portable thermal camera imaging system (FLIR T640, RS Components GmbH, Möhrfelden-Walldorf, Germany) was used during pre- and postflight BDC sessions prior to Myoton measurements (Supplementary Table S1). Cabin temperature during inflight experimental sessions was recorded from the cabin log files inside the Columbus module (between range ~ 22–24 °C).

### Ultrasound imaging

At the first BDC session, ultrasound imaging was used prior to Myoton measurement to verify anatomical location of MPs (Supplementary Figs. S2 and S3). In subsequent sessions, ultrasound followed Myoton data collection to document superficial tissue thickness (skin, subcutaneous fat, perimuscular fascia). Images were taken using a real-time B-mode ultrasound scanner (ORCHEO lite, SONOSCANNER, Paris or SUPERSONIC Aixplorer, SuperSonic Imagine, Aix-en-Provence) with a linear transducer (2–18 MHz). Inflight ultrasound sessions were performed by crew operators with a scientific team member (P.E.M., M.J.S. or M.B.W.) guiding remotely from the ground facility (CADMOS, Toulouse, France). All images were measured later off-line by one investigator (P.E.M.) using custom written Matlab (Mathworks, USA) code (written by M.B.W.). Ultrasound imaging of musculoskeletal soft tissues is a well-established valid and reliable tool, and reliability of the present protocol was demonstrated^40^.

### Baseline data collection (BDC): pre- and postflight

Anatomical location of all 10 MPs was conducted by manual palpation using easily palpable bone landmarks (e.g. acromion, vertebral spinous processes [C7, L4]), following known clinical protocols^40^. The MPs were marked on the skin using a surgical skin marker pen (Dermaskript, pmfmedical.com). Custom-made adhesive rulers (cm) were used for accurate relocation of MPs, followed by photo documentation of individual MPs to create an illustrated personalized manual as a reference for Crew members for subsequent sessions on the ISS. The participant was fully relaxed for all measurements and the first set for MP1-5 were performed in prone lying, with the hands and forearms relaxed by the sides of the body and a small pillow placed under the ankles. MP6 was measured in upright sitting, arms resting on the thighs. The participant then moved into a supine position, with a pillow under the knees, for measuring MP7-10. All MPs were only studied on the right side of the body, regardless of dominance, to minimize errors in the protocol, as it was critical to avoid wasted data. During all pre- and postflight BDC sessions, thermal FLIR camera imaging system was used to monitor skin temperature. Measurements with the MyotonPRO were then taken of MPs followed by ultrasound imaging. Before launch, astronauts received a 1 h on-the-ground training on locating anatomical structures and MPs, and using space-qualified Myoton technology and the ultrasound imaging device. Most participants were also operators inflight, so were trained. If a crew member was only a participant, they were not trained.

### Inflight data collection

According to ISS flight rules, countermeasure exercises including aerobic and resistance training were performed for up to 2–2.5 h/day for 6/week by all astronauts whilst inflight^7,45^.

Inflight Myoton data collection was performed on four different flight days (FD5-15, FD31-60, FD121-150, Return minus [R-]10 ± 5, i.e. before return to Earth) prior to daily activities (e.g. maintenance activities, onboard exercise protocols). Ultrasound imaging was performed on two flight days (FD31-60, R-10 ± 5). Data were collected by a fellow crew member (operator) using the illustrated personalized manual (Crew iPad) for reference. The participant was fully relaxed in prone (MPs 1–6) and supine (MPs 7–10) positions, with the body loosely fixed to the cabin floor with a pelvic belt to minimize movements (free-floating body shifts) to allow for passive muscle stiffness measurements. The operator used feet hooks and floor bars for their own body stabilization to enable accurate data collection from each of the skin MPs (see Supplementary Fig. S5).

### Statistical analysis

GraphPad Prism (GraphPad Prism 9.5.0 for Windows, GraphPad Software, San Diego, California, USA, www.graphpad.com) was used to process all data. Myoton data are intentionally provided as absolute values (colour-coded data clusters for each study participant for three conditions, pre/in/postflight) in scatter plots for data interpretation (at a glance) necessary for the comparison between muscle adaptation/maladaptation effects in healthy Astronauts (thus lacking pathologies or diagnosed diseases/co-morbidities usually investigated in clinical studies). Absolute values enable evaluation of the data with respect to the standard error of measurement, providing confidence in the robustness of the data, and changes in values greater than the MDC (whether increased or decreased from the mean) indicate abnormal stiffness. The Shapiro–Wilk test examined for normality of data distribution and showed a parametric distribution. Thus, one-way ANOVA with repeated measures was used, which if significant was followed by Tukey’s post hoc test, to compare passive muscle stiffness given as Newtons per metre [N/m] at different time points (preflight, inflight, postflight). Data [N/m] are presented as mean and standard deviation (SD) in 2D scatter/dot plots using participant-matched colour-coding points (Astronauts A to S) plotted against pre/in/postflight condition (circle/square/triangle data point symbols) for visualization (Figs. 1 and 2, Supplementary Fig. S1). The level of significance was set at p = 0.05 (pre/in/postflight). Changes in stiffness inflight and postflight were examined with separate linear mixed models using SPSS. (Fig. 3). Visualization of the case-wise relationship between number of days and stiffness suggested a quadratic relationship for both inflight and postflight. The quadratic was then calculated for number of days for each participant for both inflight and postflight days. The days were centered with respect to the first day inflight or first day postflight. Multiple models were developed where combinations of fixed and random effects consisting of intercept, number of days and quadratic days were used as covariates to predict the dependent variable of stiffness for each MP. The quality of fit for each model was assessed using the Akaike Information Criterion. For all measurement sites, both inflight and postflight, the best fitting model consisted of fixed effects of intercept and quadratic days, with random effect of intercept. If not indicated otherwise, MDC reference values from the 10 skin MPs were used^40^, which were produced from reliability studies involving a minimum of 20 participants.

### Ethical approval

This study followed the guidelines and regulations of the Human Research Multilateral Review Board (current HRMRB document approval notification Pro2510 Amd-7 by NASA Institutional Review Board), approved by the European Space Agency Medical Board (ESA-MB, current document approval 2023-05-02), Japan Aerospace Exploration Agency, JAXA (JX_IRBA-20-014 with document #23/JAXA/HSM No 0606001), and by Local Institutional Ethical Approval Review Board of Charité Universitätsmedizin Berlin, Germany (#EA/1/302/15), and School of Health Sciences Ethics Committee, University of Southampton UK (ERGO II #31524). Written informed consent was obtained from all participants before study inclusion. Participants were able to withdraw from the study at any time.

### Supplementary Information

Supplementary Information.

## Data Availability

The data that support the findings of this study are available from the corresponding author upon reasonable request. The data are not publicly available due to privacy regulations of ESA, NASA and JAXA.

## References

[1] Lu TW, Chang CF (2012). Biomechanics of human movement and its clinical applications. Kaohsiung J. Med. Sci..

[2] LeBlanc AD, Spector ER, Evans HJ, Sibonga JD (2007). Skeletal responses to space flight and the bed rest analog: A review. J. Musculoskelet. Neuronal Interact..

[3] Moosavi D (2021). The effects of spaceflight microgravity on the musculoskeletal system of humans and animals, with an emphasis on exercise as a countermeasure: A systematic scoping review. Physiol. Res..

[4] Juhl OJ (2021). Update on the effects of microgravity on the musculoskeletal system. NPJ Microgr..

[5] Hackney KJ (2015). The astronaut-athlete: Optimizing human performance in space. J. Strength Cond. Res..

[6] Loehr JA (2015). Physical training for long-duration spaceflight. Aerosp. Med. Hum. Perform..

[7] Scott JPR, Weber T, Green DA (2019). Editorial: Optimization of exercise countermeasures for human space flight-lessons from terrestrial physiology and operational implementation. Front. Physiol..

[8] Ford JJ (2020). Effects of specific muscle activation for low back pain on activity limitation, pain, work participation, or recurrence: A systematic review. Musculoskelet. Sci. Pract..

[9] Stokes, M. *et al.* Recommendations for Future Post-mission Neuro-Musculoskeletal Reconditioning Research and Practice Post-mission Exercise (Reconditioning) Topical Team Report. (2016).

[10] Worth Jr, M. H., Manning, F. J. & Longnecker, D. E. Review of NASA's longitudinal study of astronaut health. (2004). 25009858

[11] Lee IM (2012). Effect of physical inactivity on major non-communicable diseases worldwide: An analysis of burden of disease and life expectancy. Lancet.

[12] Thomas E (2019). Physical activity programs for balance and fall prevention in elderly: A systematic review. Medicine (Baltimore).

[13] Graham ZA (2021). Mechanisms of exercise as a preventative measure to muscle wasting. Am. J. Physiol. Cell Physiol..

[14] Garrett-Bakelman FE (2019). The NASA twins study: A multidimensional analysis of a year-long human spaceflight. Science.

[15] Trappe S (2002). Effects of spaceflight, simulated spaceflight and countermeasures on single muscle fiber physiology. J. Gravit. Physiol..

[16] Colleran PN (2000). Alterations in skeletal perfusion with simulated microgravity: A possible mechanism for bone remodeling. J. Appl. Physiol..

[17] Baskin KK, Winders BR, Olson EN (2015). Muscle as a "mediator" of systemic metabolism. Cell Metab..

[18] Edgerton VR (2001). Sensorimotor adaptations to microgravity in humans. J. Exp. Biol..

[19] Drummer C (2000). Regulation and distribution of body fluid during a 6-day head-down tilt study in a randomized cross-over design. J. Gravit. Physiol..

[20] Schleip R (2006). Passive muscle stiffness may be influenced by active contractility of intramuscular connective tissue. Med. Hypotheses.

[21] Briguglio M (2021). Nutritional orthopedics and space nutrition as two sides of the same coin: A scoping review. Nutrients.

[22] Lambertz D, Pérot C, Kaspranski R, Goubel F (2001). Effects of long-term spaceflight on mechanical properties of muscles in humans. J. Appl. Physiol..

[23] Bartsch K (2023). Assessing reliability and validity of different stiffness measurement tools on a multi-layered phantom tissue model. Sci. Rep..

[24] Mijailovic AS, Qing B, Fortunato D, Van Vliet KJ (2018). Characterizing viscoelastic mechanical properties of highly compliant polymers and biological tissues using impact indentation. Acta Biomater..

[25] Masi AT, Hannon JC (2008). Human resting muscle tone (HRMT): Narrative introduction and modern concepts. J. Bodyw. Mov. Ther..

[26] Simons GD, Mense S (1998). Understanding and measurement of muscle tone as related to clinical muscle pain. Pain.

[27] Agyapong-Badu S, Warner M, Samuel D, Stokes M (2018). Practical considerations for standardized recording of muscle mechanical properties using a myometric device: Recording site, muscle length, state of contraction and prior activity. J. Musculoskelet. Res..

[28] Bernabei M, Lee SS, Perreault EJ, Sandercock TG (2020). Shear wave velocity is sensitive to changes in muscle stiffness that occur independently from changes in force. J. Appl. Physiol..

[29] Agyapong-Badu S, Warner M, Samuel D, Stokes M (2016). Measurement of ageing effects on muscle tone and mechanical properties of rectus femoris and biceps brachii in healthy males and females using a novel hand-held myometric device. Arch. Gerontol. Geriatr..

[30] Dankel SJ, Razzano BM (2020). The impact of acute and chronic resistance exercise on muscle stiffness: A systematic review and meta-analysis. J. Ultrasound.

[31] Yu HK (2022). Performance of passive muscle stiffness in diagnosis and assessment of disease progression in duchenne muscular dystrophy. Ultrasound Med. Biol..

[32] Rätsep T, Asser T (2011). Changes in viscoelastic properties of skeletal muscles induced by subthalamic stimulation in patients with Parkinson's disease. Clin. Biomech. (Bristol, Avon).

[33] Svantesson U, Takahashi H, Carlsson U, Danielsson A, Sunnerhagen KS (2000). Muscle and tendon stiffness in patients with upper motor neuron lesion following a stroke. Eur. J. Appl. Physiol..

[34] Demangel R (2017). Early structural and functional signature of 3-day human skeletal muscle disuse using the dry immersion model. J. Physiol..

[35] Schneider S, Peipsi A, Stokes M, Knicker A, Abeln V (2015). Feasibility of monitoring muscle health in microgravity environments using Myoton technology. Med. Biol. Eng. Comput..

[36] Technology - Myoton. (2023).

[37] Lee Y, Kim M, Lee H (2021). The measurement of stiffness for major muscles with shear wave elastography and myoton: A quantitative analysis study. Diagnostics.

[38] Chuang LL, Wu CY, Lin KC, Lur SY (2012). Quantitative mechanical properties of the relaxed biceps and triceps brachii muscles in patients with subacute stroke: A reliability study of the myoton-3 myometer. Stroke Res. Treat..

[39] García-Bernal MI, González-García P, Madeleine P, Casuso-Holgado MJ, Heredia-Rizo AM (2023). Characterization of the structural and mechanical changes of the biceps brachii and gastrocnemius muscles in the subacute and chronic stage after stroke. Int. J. Environ. Res. Public Health.

[40] Muckelt PE (2022). Protocol and reference values for minimal detectable change of MyotonPRO and ultrasound imaging measurements of muscle and subcutaneous tissue. Sci. Rep..

[41] Jarocka E, Marusiak J, Kumorek M, Jaskólska A, Jaskólski A (2011). Muscle stiffness at different force levels measured with two myotonometric devices. Physiol. Meas..

[42] Hu X (2018). Quantifying paraspinal muscle tone and stiffness in young adults with chronic low back pain: A reliability study. Sci. Rep..

[43] Bierman W (1936). The temperature of the skin surface. JAMA.

[44] Yoon S-W, Lee J-W, Kim M-J, Kim S-H, Park W-S (2012). A study of muscular activities and onset times of the tibialis anterior and medial gastrocnemius muscles of elderly people in climbing stairs. J. Phys. Ther. Sci..

[45] Petersen N (2016). Exercise in space: The European Space Agency approach to in-flight exercise countermeasures for long-duration missions on ISS. Extrem Physiol. Med..

[46] Fitts RH (2010). Prolonged space flight-induced alterations in the structure and function of human skeletal muscle fibres. J. Physiol..

[47] Koryak YA (2019). Changes in human skeletal muscle architecture and function induced by extended spaceflight. J. Biomech..

[48] Rubenson J, Pires NJ, Loi HO, Pinniger GJ, Shannon DG (2012). On the ascent: The soleus operating length is conserved to the ascending limb of the force-length curve across gait mechanics in humans. J. Exp. Biol..

[49] Kubo K (2004). Effects of 20 days of bed rest on the viscoelastic properties of tendon structures in lower limb muscles. Br. J. Sports Med..

[50] Schoenrock B (2018). Bed rest, exercise countermeasure and reconditioning effects on the human resting muscle tone system. Front. Physiol..

[51] Labonte D, Holt NC (2022). Elastic energy storage and the efficiency of movement. Curr. Biol..

[52] Obst SJ, Newsham-West R, Barrett RS (2016). Changes in Achilles tendon mechanical properties following eccentric heel drop exercise are specific to the free tendon. Scand. J. Med. Sci. Sports.

[53] Chang TT (2020). Objective assessment of regional stiffness in achilles tendon in different ankle joint positions using the MyotonPRO. Med. Sci. Monit..

[54] Magnusson SP, Narici MV, Maganaris CN, Kjaer M (2008). Human tendon behaviour and adaptation, in vivo. J. Physiol..

[55] Lorimer AV, Hume PA (2016). Stiffness as a risk factor for achilles tendon injury in running athletes. Sports Med..

[56] Holm C, Kjaer M, Eliasson P (2015). Achilles tendon rupture–treatment and complications: A systematic review. Scand. J. Med. Sci. Sports.

[57] Morgan GE, Martin R, Williams L, Pearce O, Morris K (2018). Objective assessment of stiffness in Achilles tendinopathy: A novel approach using the MyotonPRO. BMJ Open Sport Exerc. Med..

[58] Hora M, Struška M, Matějovská Z, Kubový P, Sládek V (2023). Muscle activity during crouched walking. Am. J. Biol. Anthropol..

[59] Nakamura M (2021). Relationship between changes in passive properties and muscle strength after static stretching. J. Bodyw. Mov. Ther..

[60] Brower RG (2009). Consequences of bed rest. Crit. Care Med..

[61] Penchev R (2021). Back pain in outer space. Anesthesiology.

[62] Bailey JF (2018). From the international space station to the clinic: how prolonged unloading may disrupt lumbar spine stability. Spine J..

[63] Plehuna A (2022). Dry immersion induced acute low back pain and its relationship with trunk myofascial viscoelastic changes. Front. Physiol..

[64] Green DA, Scott JPR (2017). Spinal health during unloading and reloading associated with spaceflight. Front. Physiol..

[65] Noonan AM, Brown SHM (2021). Paraspinal muscle pathophysiology associated with low back pain and spine degenerative disorders. JOR Spine.

[66] Patel ZS (2020). Red risks for a journey to the red planet: The highest priority human health risks for a mission to Mars. NPJ Microgr..

[67] Beaudart C (2019). Assessment of muscle function and physical performance in daily clinical practice : A position paper endorsed by the European Society for Clinical and Economic Aspects of Osteoporosis, Osteoarthritis and Musculoskeletal Diseases (ESCEO). Calcif Tissue Int..

[68] Bailey JF (2022). Biomechanical changes in the lumbar spine following spaceflight and factors associated with postspaceflight disc herniation. Spine J..

[69] Basti A (2021). Diurnal variations in the expression of core-clock genes correlate with resting muscle properties and predict fluctuations in exercise performance across the day. BMJ Open Sport Exerc. Med..

[70] Wu Z (2021). Effects of age and sex on properties of lumbar erector spinae in healthy people: Preliminary results from a pilot study. Front. Physiol..

[71] Marusiak J, Jaskólska A, Koszewicz M, Budrewicz S, Jaskólski A (2012). Myometry revealed medication-induced decrease in resting skeletal muscle stiffness in Parkinson's disease patients. Clin. Biomech..

[72] Agoriwo MW (2022). Feasibility and reliability of measuring muscle stiffness in Parkinson’s Disease using MyotonPRO device in a clinical setting in Ghana. Ghana Med. J..

[73] Gunga H-C (2020). Human Physiology in Extreme Environments.

[74] Qian L, Zhao H (2018). Nanoindentation of soft biological materials. Micromachines (Basel).

