# Supplementary material for: Muscle stiffness indicating mission crew health in space
Source: Sci Rep. 2024 Feb 20;14:4196. doi: 10.1038/s41598-024-54759-6 (PMC10879143; doi:10.1038/s41598-024-54759-6)
Supplement: Supplementary file 1 — Supplementary Information. [file 41598_2024_54759_MOESM1_ESM.pdf]

Muscle stiffness indicating mission crew health in space

Britt Schoenrock, Paul E. Muckelt, Maria Hastermann, Kirsten Albracht, Robert MacGregor, David Martin, Hans-Christian Gunga, Michele Salanova, Maria J. Stokes, Martin B. Warner, Dieter Blottner

MYOTONES data from additional skin measurement points

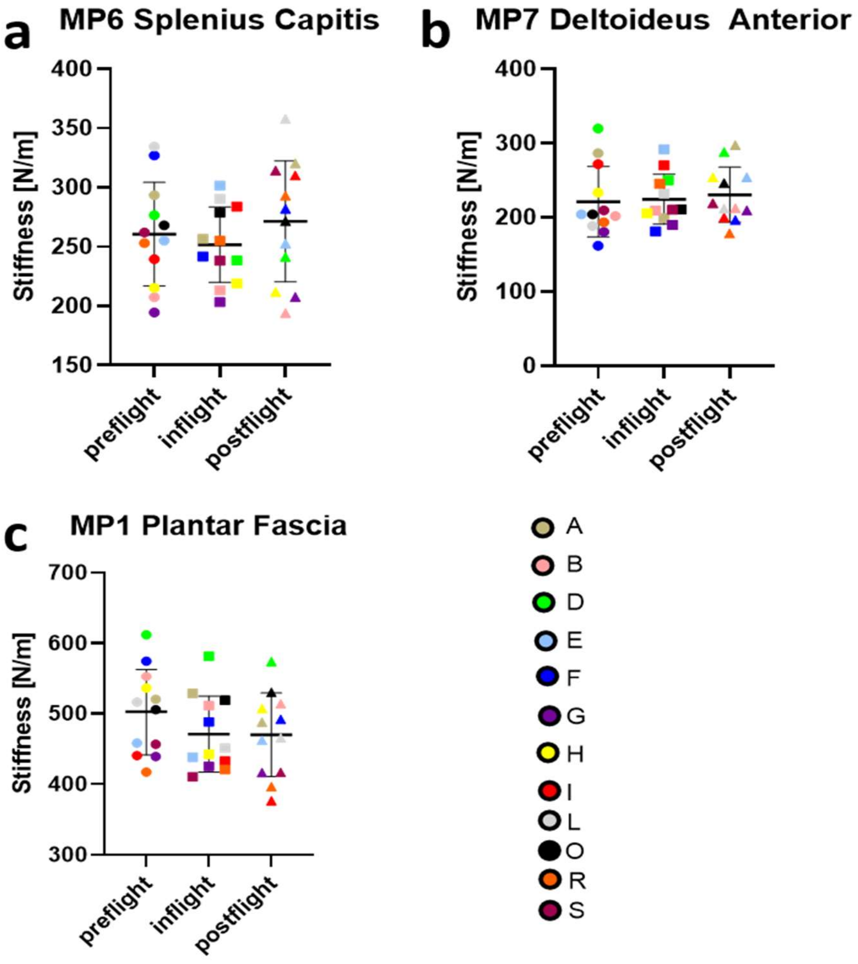

Supplementary Figure S1. Stiffness MYOTONES data from remaining sites not shown in Figs 1 and 2 within manuscript. a, MP6 Splenius Capitis (deep long neck muscle); **b**, Deltoideus anterior (anterior part of shoulder muscle); **c**, Plantar Fascia (foot sole superficial ligament). No statistical difference found in these structures apart from intersubject variability (see colour codes A-S) during the missions. Mean of n=12 (for each test condition) with standard deviation for preflight (BDC L-180 and BDC L-60), inflight (FD5-15, FD30-60, FD121-150 and, R-10) and postflight (R+1, R+5, R+30, and R+105)

Linear mixed model data for significant parameters

Supplementary Table S1. Linear mixed model results for measurement sites that had significant parameters for both inflight and postflight phases of the study. All parameters are fixed effects. 'Quad-days' is the number of days transformed into quadratic form to account for the curvilinear relationship observed between stiffness and number of days spent inflight or postflight.

Measurement site	Parameter	Estimate	Std. Error	df	t	Sig.	95% Confidence Interval	
							Lower Bound	Upper Bound
**Inflight**								
**Plantar Fascia (MP1)**	Intercept	485.2	16.6	12.7	29.2	P<0.001	449.3	521.2
	Quad-days	-1.3	0.4	35.2	-3.3	P=0.002	-2.0	-0.5
**Achilles Tendon (MP2)**	Intercept	633.9	17.8	12.3	35.6	P<0.001	595.2	672.5
	Quad-days	-0.8	0.4	35.2	-2.2	P=0.036	-1.5	-0.1
**Soleus (MP3)**	Intercept	342.3	8.1	11.4	42.4	P<0.001	324.6	359.4
	Quad-days	0.4	0.2	32.2	2.5	P=0.02	0.08	0.8
**Patellar Tendon (MP9)**	Intercept	720.9	28.9	14.0	25.0	P<0.001	658.9	782.8
	Quad-days	-1.8	0.9	35.3	-2.1	P=0.04	-3.6	-0.1
**Postflight**								
**Plantar Fascia (MP1)**	Intercept	466.3	16.7	11.6	28.0	P<0.001	429.9	502.8
	Quad-days	1.6	0.8	35.1	2.0	P=0.05	0.003	3.2
**Anterior Deltoid (MP7)**	Intercept	235.9	11.3	12.1	20.4	P<0.001	210.6	260.0
	Quad-days	-1.8	0.8	35.2	-2.4	P=0.02	-3.4	-0.3
**Patellar Tendon (MP9)**	Intercept	618.5	34.8	11.4	17.8	P<0.001	542.2	694.8
	Quad-days	3.0	1.30	35.1	2.3	P=0.03	0.3	5.7

Std Error = Standard error; df = degrees of freedom; t = t value; sig. = significance value.

On the ground surface skin temperature results

Supplementary Table S2. On the ground surface skin temperature (°C) of Astronauts at MYOTONES skin measurement points MP1 to 10 (first column) during pre (L-180/-60) and postflight (R+1/5/30/105) measurement sessions. Pooled data (mean) from n=12 Astronauts for each time point given as days before launch (L-180/-60) to Space, and days after return (R+1/5/30/105) back to Earth.

MP	L-180	L-60	R+1	R+5	R+30	R+105
**MP1**	29.65*	29.02	30.78	30.24	29.9	30.18
**MP2**	29.58	30.23	30.08	29.94	29.6	29.82
**MP3**	31.78	32.57	32.41	32.31	32.33	32.95
**MP4**	32.53	33.1	32.57	32.56	32.69	32.81
**MP5**	34.41	34.65	33.92	33.86	34.13	34.16
**MP6**	34.15	35.38	35.18	34.78	34.56	34.82
**MP7**	33.75	33.7	33.63	33.52	33.53	33.51
**MP8**	33.09	33.55	3302	32.69	32.83	32.84
**MP9**	31.06	31.32	30.91	30.89	30.93	30.63
**MP10**	32.45	32.58	32.33	31.95	32.52	32.44

*Numbers represent degrees centigrade (°C) recorded by FLIR ThermoCam technology.

Subcutaneous tissue thickness in Astronauts for all skin measurement points before, during and after spaceflight

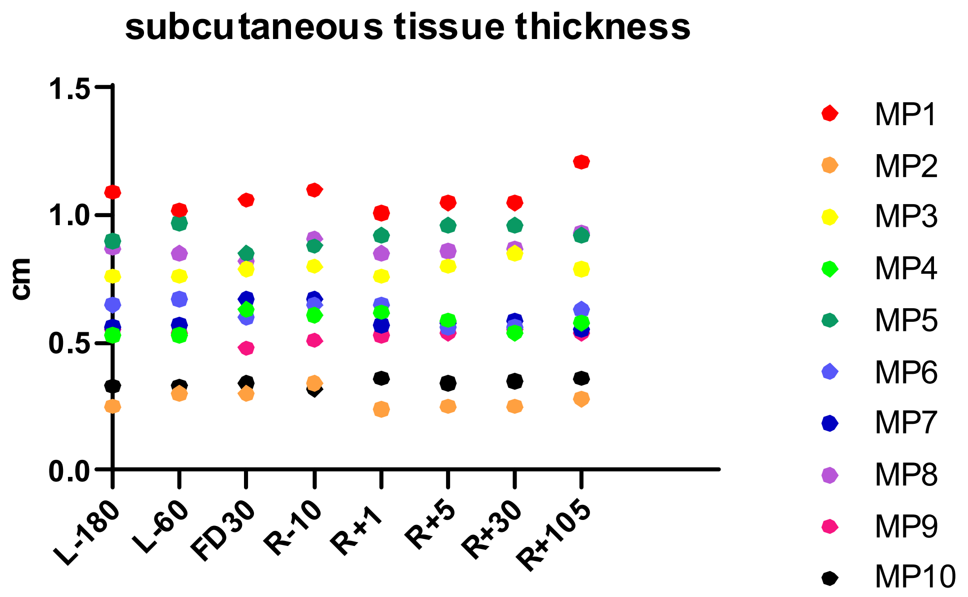

Supplementary Figure S2. Subcutaneous tissue thickness in Astronauts (Mean of n=12) for all 10 MPs over time (sessions). Ultrasound measurements were taken at two preflight session days (L-180, L-60), two inflight (FD30, R-10) and four postflight session days (R+1 to R+105). Colour codes represent individual skin measurement points, MPs (pooled data from n=12 astronauts).

Representative ultrasound images from the thigh Quadriceps muscle to show subcutaneous thickness determination

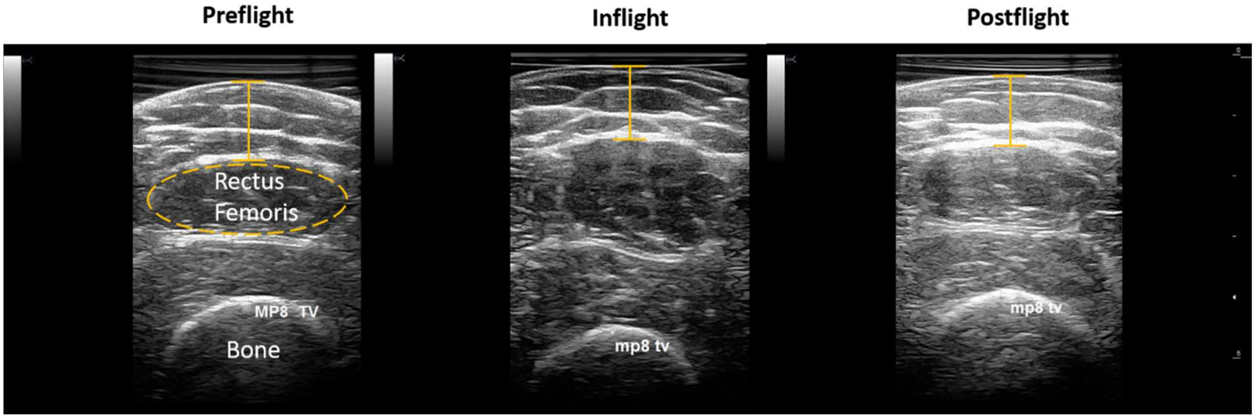

Supplementary Figure S3. Subcutaneous thickness in an Astronaut thigh muscle (Quadriceps femoris). Three axial (transverse, TV) ultrasound images from Quadriceps (with Rectus Femoris head, oval orange broken line) beneath the skin measurement point #8 (MP8). Vertical yellow brackets denote subcutaneous thickness for each condition (pre-in-postflight). See Supplementary Figure S2 for all MP indices (in cm).

MYOTONES experimental design

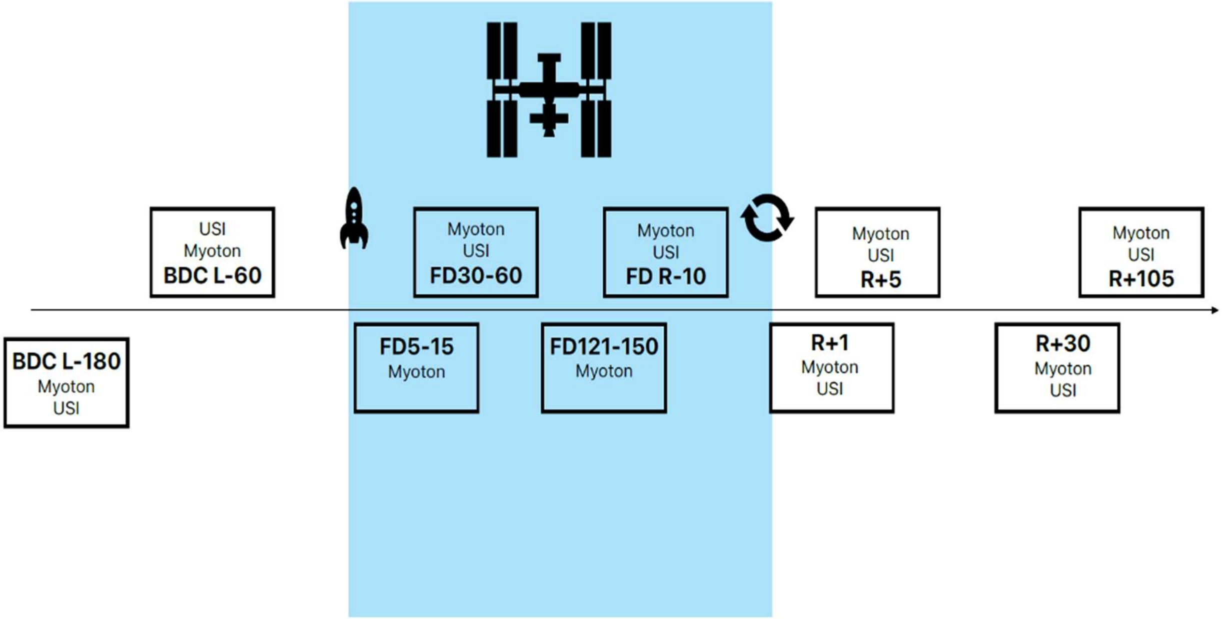

Supplementary Figure S4. Timeline of MYOTONES experiment. Myoton stiffness data collection was performed on two preflight baseline data collection (BDC) days before launch (BDC L-), four inflight days (periods of time window; FD), and four postflight baseline data collection measurements on return/landing day (R+1) and follow-up return/recovery days (R+5/30/105) with Myoton and remote ultrasound imaging (USI). Blue box highlights inflight FD sessions on ISS.

MYOTONES body chart of the ten skin measurement points on Astronauts

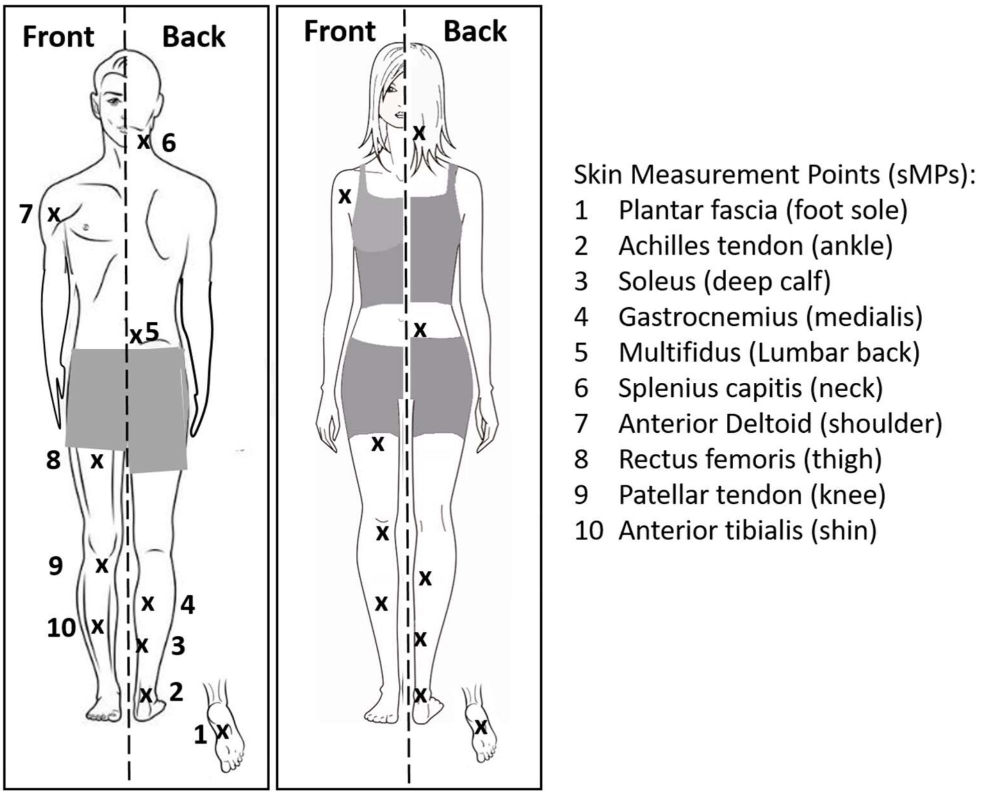

Supplementary Figure S5. Myoton protocol body chart (male/female) of the 10 skin measurement points (MPs). Skin MPs included one fascia (MP1), two tendons (MP2 and MP9) and seven muscles (MP3, 4, 5, 6, 7, 8 and 10). MP1-6 in supine position (back view), MP7-10 in prone position (front view).
